# HTLV-1 infected CD4+T cells of HAM/TSP patients are susceptible to tunicamycin-induced endoplasmic reticulum stress

**DOI:** 10.1186/1742-4690-12-S1-P19

**Published:** 2015-08-28

**Authors:** Daisuke Kodama, Ryuji Kubota, Toshio Matsuzaki, Hiroshi Takashima, Shuji Izumo

**Affiliations:** 1 Molecular Pathology, Center for Chronic Viral Diseases, Kagoshima University Graduate School of Medical and Dental Sciences. Kagoshima-Shi, Kagoshima-Ken, 890-8544, Japan; 2 Medical Corporation Sanshukai Ohkatsu Hospital, Kagoshima-Shi, Kagoshima-Ken, 890-67, Japan; 3 Department of Neurology, Kagoshima University Graduate School of Medical and Dental Sciences. Kagoshima-Shi, Kagoshima-Ken, 890-8520, Japan

## 

Lectin array analysis for membrane proteins from CD4+Tcells of four respective cases of HAM, healthy carriers (HC), and negative controls (NC) showed significant high signal for Urtica dioica agglutinin (UDA) and Solanum tuberosum (Potato) (STL) lectins, that specifically recognize N-glycan, N-acetyllactosamine (LacNAc) which consist of repeats of the disaccharide βGal(1-4)βGlcNAc(1-3). We verified that LacNAc is significantly expressed in CD4+T cells in HAM by lectin ELISA for each ten cases of HAM, HC, and NC, followed by qRT-PCR of β1,3-N-acetylglucosaminyltransferase 2 (B3GNT2) gene which was reported as main responsible enzyme in LacNAc biosynthesis. Using tunicamycin(TM), known as a N-glycan inhibitor and an apoptosis inducing agent through endoplasmic reticulum (ER) stress, we investigated the effect of LacNAc suppression on CD4+T cells. In NC, both of LacNAc+ and LacNAc-cells showed similar survival regardless of treatment with TM of 2μM for 24h or without TM, however, both cells in HAM showed worse survival than in NC and LacNAc+cells showed better survival when treated with TM. Moreover in HAM, Tax-cells showed better survival regardless of treatment with TM and LacNAc+cells when with treatment with TM survived significantly better than when without TM. Tax+cells showed worse survival than Tax-cells and tended to decrease when treated with TM. Collectively, we hypothesized that LacNAc is a resistant factor to TM-induced apoptosis in HTLV-1 uninfected cells but HTLV-1 infected cells may be susceptible to TM even if LacNAc are expressed. To confirm this hypothesis, we performed qRT-PCR of main effector genes in ER stress pathway: x-box binding protein 1, spliced variant (XBP1s), heat shock 70kDa protein 5(GRP78), and heat shock protein 90kDa beta, member 1(GRP94). Only GRP94 was significantly suppressed in HAM and HC. We conclude that HTLV-1 infected cells may be under ER stress and be susceptible to TM, and that LacNAc positive cells are resultant survivors.

